# A high-risk group of pregnant women with elevated levels of conflict-related trauma, intimate partner violence, symptoms of depression and other forms of mental distress in post-conflict Timor-Leste

**DOI:** 10.1038/tp.2015.212

**Published:** 2016-02-02

**Authors:** S J Rees, W Tol, M Mohammad, A K Tay, N Tam, N dos Reis, E da Costa, C Soares, D M Silove

**Affiliations:** 1 Psychiatry Research and Teaching Unit, University of New South Wales and Academic Mental Health Unit, Mental Health Centre, Liverpool Hospital, Sydney, NSW, Australia; 2 Bloomberg School of Public Health, Johns Hopkins University, Baltimore, MD, USA; 3 Child and Maternal Health, Alola Women's Foundation, Dili, Timor-Leste

## Abstract

Women in post-conflict, low-income, post-conflict (LI-PC) countries are at risk of exposure to the traumatic events (TEs) of war and intimate partner violence (IPV), forms of stress that are known to lead to depression and other adverse mental health outcomes. We aimed to assess an index of exposure to these two forms of trauma to identify pregnant women attending antenatal clinics in conflict-affected Timor-Leste at high risk of depression and other forms of stress. A large, cross-sectional study of women in the second trimester of pregnancy was conducted in the four main government antenatal clinics in Dili district of Timor-Leste, between May 2014, and January 2015. The sample consisted of 1672 consecutive women, 3 to 6 months pregnant, with a response rate of 96%. We applied the Edinburgh Postnatal Depression Scale, the Kessler-10 psychological distress scale and the Harvard Trauma Questionnaire. IPV was assessed by the World Health Organisation measure. Composite categories of conflict-related TEs and severity of IPV showed a dose–response relationship with depressive symptoms: for exposure to four or more conflict-related TEs and severe psychological IPV, the adjusted odds ratio (AOR) was 3.95 (95% confidence interval (CI) 2.10–7.40); for four or more TEs and physical abuse, AOR 8.16 (95% CI 3.53–18.85); and for four or more TEs and severe psychological and physical abuse, AOR 9.78 (95% CI 5.31–18.02). For any mental distress, the AOR for four or more TEs and severe psychological abuse was 3.60 (95% CI 2.08–6.23); for four or more TEs and physical abuse 7.03 (95% CI 3.23–15.29); and for four or more TEs and severe psychological and physical abuse the AOR was 10.45 (95% CI 6.06–18.01). Of 184 women (11% of the sample) who reported ⩾4 TEs and either physical abuse alone or in combination with severe psychological abuse, 78 (42%) reached threshold for depressive symptoms and 93 (51%) for any mental distress, a 10-fold increase in depressive and other mental health symptoms. Priority should be directed to providing urgent mental health and social interventions for this group of women. Our findings offer a framework for a tiered approach to detection, guiding prevention and intervention strategies for IPV and associated mental health problems in low-income post-conflict countries.

## Introduction

High-fertility rates in low-income countries result in women spending a considerable time in pregnancy.^1^ In low-income countries that have experienced mass conflict, women are at risk of exposure to both the traumatic events (TEs) of warfare and intimate partner violence (IPV),^2, 3, 4, 5^ stressors that may increase risk of antenatal depression and related forms of mental distress. An important question is whether a combined index based on the severity of exposure to these two forms of trauma (conflict-related TEs and IPV) can assist in identifying pregnant women in greatest need of interventions designed to address their ongoing mental health problems and risk of exposure to further violence in the home. We report the largest study undertaken worldwide of pregnant women in a low-income, post-conflict (LI-PC) country (Timor-Leste) which aims to address these issues.

There are compelling reasons to focus on the TEs of conflict and IPV among pregnant women in low-income countries. A strong relationship has been established between IPV and depression among women in high-income countries, and evidence of a comparable association is emerging in low and middle income countries (LMICs),^6, 7, 8^ particularly those exposed to mass conflict.^5, 9^ General population studies show that past exposure to conflict-related TEs increases risk of depression and post-traumatic stress disorder (PTSD).^10^ Women are at increased risk of both disorders, and pregnancy is a period of heightened vulnerability to depression in particular.^6^ High fertility rates in LI-PC countries result in a large portion of women in early and mid-adulthood spending a considerable time in pregnancy, thereby increasing the period of risk for depression.^1^ A considerable number of pregnant women in LI-PC countries have been exposed to past TEs of conflict and IPV, making it important to distinguish those in the highest risk group for depression and other stress-related mental disorders, to ensure that limited resources are directed to those with the greatest need of intervention. To facilitate this task, it is vital to develop and test a hierarchical typology based on combined exposure to TEs of conflict and IPV to identify a putatively high-risk group of women requiring priority interventions focusing on their mental health problems and need for protection from further violence.^11^

There is a remarkable dearth of data focusing on the mental health of pregnant women in LI-PC countries. A meta-analysis of studies undertaken in LMICs as a whole confirmed a high rate of common mental disorders in pregnancy, but only a limited number of these studies were conducted in LI-PC countries.^6^ The seven studies in LMICs that included an index of IPV showed a consistent association between that form of abuse and risk of common mental disorders, in one instance, conforming to a dose–response relationship.^12^ Past studies in LMICs, however, have included less than 200 participants and none has investigated whether the combination of exposure to the TEs of conflict and IPV identifies women with the most severe mental health outcomes.

One challenge faced by past studies has been the difficulty of disaggregating the effects of physical IPV from psychological IPV, largely because the two forms of abuse frequently co-occur. Psychological abuse as defined by most standardised measures is also widely experienced, particularly in countries that promote and maintain traditional gendered norms. Excluding psychological abuse from analyses however, a strategy applied in several large studies,^13, 14, 15^ risks ignoring an important contributor to adverse mental health outcomes, an issue highlighted in studies in high-income countries.^16, 17, 18, 19^ As yet, therefore, the impact of psychological abuse on the mental health of women in LI-PC settings remains to be clarified.^6^ In our study, we derived a novel typology based on combinations of physical and psychological forms of abuse ordered according to a hierarchy of severity.^20^

Timor-Leste is an exemplar of a contemporary LI-PC, our study being timely in that it was conducted at a point when women in the child-bearing age had lived through both major periods of mass conflict in the country. During the Indonesian occupation (1975–1999) and subsequent humanitarian emergency (1999–2000), the population was exposed to extensive human rights violations including murder, torture, arbitrary incarceration and mass displacement.^21^ Systematic violations that were commonly perpetrated against women included rape, forced marriage and sexual harassment.^22^ A vote on independence in 1999 was followed by a humanitarian emergency, the United Nations assuming control of the territory to restore peace which was largely maintained through the period of national independence. There was a further period of mass violence in 2006–2007, involving warring factions within the country, resulting in mass displacement of populations, injuries and further destruction of property.^23^ In addition, consistent with other countries in the geographical region, IPV rates are high in Timor-Leste, estimates indicating that 38% of women experience physical abuse by an intimate partner after age 15 years.^24^

Our broad objective is to apply a typology of trauma that includes increasing levels of severity of the TEs of conflict and IPV to identify pregnant women at ultra-high risk of symptoms of depression and other forms of mental distress. We focus first on testing associations between escalating levels of severity of IPV and mental health indices. We then apply our composite index comprising combinations of escalating severity of TEs of conflict and IPV to examine patterns of association with depressive symptoms and other adverse mental health outcomes. The overall objective is to identify an ultra-high-risk group of antenatal women warranting priority intervention.

## Materials and methods

### Study setting

The long-standing partnership of our research team with the Alola Foundation, the peak women's non-government organization in Timor-Leste, facilitated the process of engagement with all government perinatal services in the Dili district, a rural and urban administrative area resident to 21.9% of the total Timorese population of 1.1 million persons. Government statistics indicated that 96% of women in the Dili district received at least one occasion of antenatal care from a skilled professional during pregnancy,^24^ confirming that sampling from these clinics would include the large majority of eligible women. We implemented a rolling recruitment strategy, our field team spending 3–4-month periods in succession at each of the four large government antenatal clinics (Becora, Bairo Pite/Vera Cruz, Comoro and Formosa) in the district, data collection spanning the period May 2014 to January 2015. Excluded were the small minority of women who attended private clinics, and for logistic reasons women attending two small remote community antenatal clinics (comprising 5.7% of pregnant women in the Dili district).

### Sample

Data from a preliminary study (*n*=427) undertaken in adjacent districts indicated that 400 participants were required to generate stable prevalence estimates for each clinic.^25^ We sampled consecutive attenders in the second trimester (3–6 months) of pregnancy, the peak period of registration at antenatal clinics. Excluded were women with overt psychosis, profound intellectual impairment or severe medical illness requiring referral to hospital.

### Procedure and personnel

All consecutive women who were eligible were approached at intake, the majority providing consent and being interviewed in a private space at the clinic. Four percent elected to complete interviews at a later home visit. Field staff comprised Timorese women with extensive training and experience in community-based mental health research based on their long-term involvement in the team's previous studies. Following established practice, we provided participating women with a package of soap to compensate for their time.

### Data management

Surveys were checked for omissions or out of range values immediately following interview and again at the research office prior to being entered in de-identified form by a trained data assistant into a password protected computer. Further systematic checking was undertaken of electronic files. Two records were excluded from the analysis because of missing data (more than one item). Original surveys were stored in a locked cabinet in the research office prior to being transferred to the University of New South Wales (UNSW) in Sydney for secure storage.

### Survey measures

Socio-demographic data were recorded using items from the national census, including age, sex, marital status, education, schooling and employment history.

We selected the most widely used and tested measures in the field to assess key indices of IPV and mental health. International guidelines were applied in the cultural and linguistic adaptation, translation and back-translation of measures.^26^ Final checking was undertaken by a committee comprising both expatriate experts and members of the local community. We use international cut-offs for mental health symptom measures to facilitate comparison of findings with studies conducted in other countries.

### Mental health measures

For depression, we used a culturally adapted and tested version of the Edinburg Depression Scale (EDS) which comprises 10 items scored on a 4-point scale (the summary score ranging from 0 to 30). We report the international cut-off score (>13) as an indicative threshold for depressive symptoms. To assess PTSD symptoms, we applied the 16 items of the Harvard Trauma Questionnaire, scored on a 4-point frequency scale. The summary score^1, 2, 3, 4^ is calculated by dividing the total score by the number of items. We report the community cut-off for PTSD symptoms of 2.0.^27^ As a general measure of distress, we used the Kessler-10 scale, applying the international cut-off of >30 as an index of psychological distress.

### Traumatic events

We previously modified the TE list of the Harvard Trauma Questionnaire to ensure its relevance to Timor-Leste.^28^ The list comprises conflict-related TEs (including torture, combat, conflict-related injury, witnessing murder and atrocities against family and others), as well as a few items recording exposure to natural disasters (flood and earthquake) which are common in Timor-Leste. In our past studies in Timor-Leste, we found that the majority of the population endorsed at least one trauma and that this level of exposure makes a negligible contribution to risk of mental disorder, a finding now confirmed in studies undertaken at an international level.^29^ For that reason, to maximise statistical power, the reference group for our analysis comprised participants exposed to either no or one TE. Based on the distribution of scores, the remainder was assigned to categories of two to three or four or more TEs.

### Intimate partner violence

We applied the physical and psychological abuse items of the WHO Multi-Country Study on Women's Health and Domestic Violence measure which has been applied across 14 countries worldwide.^14^ The timeframe was the 12-month prevalence of IPV enacted by the current or most recent partner. Our experience in piloting combined with advice from cultural consultants led us to exclude the sexual abuse items because the topic was highly sensitive for women in a traditional and deeply religious society. Based on considerations of the distribution and overlap of the psychological and physical domains of IPV, we assigned women to five hierarchically ordered categories: (1) No abuse (no IPV items endorsed); (2) Low respect/regard (whether spouse spends free time with the respondent, consults her on household matters; respects her and her wishes; trusts her with money); (3) Severe psychological abuse (jealous or angry if she talks to other men; frequent accusations of being unfaithful; does not permit meetings with girl friends; limits contact with family; insists on knowing woman's whereabouts; humiliates her in front of others; threatens harm to partner/someone close; lack of affection). (4) Physical abuse alone (pushing, shaking or throwing items; slapping, twisting arm; punching; kicking/dragging; strangling/burning; threats with knife, gun or other weapon; attacks with a knife, gun or other type of weapon; other forms of physical abuse). (5) Combination of physical abuse and severe psychological abuse. Assignment of women to the higher level categories (3–5) was not dependent on whether or not they also endorsed items from category 1 (low respect/regard).

### Ethics

The Human Research Ethics Committee of the UNSW and the National Health Research Cabinet of Timor-Leste approved the study. Women were informed verbally of the nature of the study because of low-literacy levels. They either signed or marked translated consent forms in the presence of a witness.

### Statistical analyses

We conducted a series of multiple logistic regression models. The first analysis examined for associations between IPV categories and the mental health indices of depression, PTSD and psychological distress, as well as a composite index of any mental distress category (that is, where participants reached threshold for at least one of the three mental health indices). Given there were no statistical differences between the no abuse (category 1) and low respect/regard (category 2) of IPV, the two were combined as a reference category, thereby increasing statistical power. In the second multivariate logistic regression analysis, we applied the comprehensive typology to categorise women according to combinations of severity of TEs of conflict (two to three; four or more) and IPV, the reference category being women reporting the combined pattern of no to one TEs and the composite low IPV categories of no abuse or low regard/respect.

Multivariate analyses controlled for socio-demographic variables (age, marital status, educational attainment and employment), clustering by clinic and other predictor variables in the model. The results are reported as adjusted odds ratios (AORs) with 95% confidence intervals (CIs). Statistical differences were reported as *P*<0.05* and *P*<0.01**. Given the low rate of missing data, we removed the two cases in which one item of a mental health measure was omitted. The analysis was performed using IBM SPSS version 22 (Armonk, NY, USA).^30^

## Results

Table 1 shows that among the full sample of 1672 women, almost three quarters (72.7%, *n*=1215) reported exposure to ⩾2 conflict-related TEs. Based on our typology of IPV, 180 (10.8%) women were assigned to the no IPV category, a third (*n*=550; 32.9%) to the low regard/respect category alone; 30.6% (*n*=511) to the severe psychological abuse category; 1 in 20 (6.2%, *n*=103) to the physical abuse category; and almost one-fifth (*n*=327) to the combined severe psychological and physical abuse category (Table 1).

One in five women (19.7%) met threshold for depressive symptoms on the EDS, one in 20 (5.7%) for PTSD symptoms and a similar portion (6.3%) for severe psychological distress. A quarter (24.1%) fell into the any mental distress category, reaching symptom threshold for at least one of the three mental health indices measured (Table 1).

In reporting further results, we include only statistically significant associations expressed as AORs, the relevant CIs being indicated in tables. Table 2 shows a regular dose–response relationship between the hierarchically ordered IPV categories and mental health indices. Compared with the combined categories of no abuse and low regard/respect only, the severe psychological abuse category was associated with both depressive symptoms (AOR 1.61) and any mental distress (1.67). The associations were even stronger for physical abuse alone (for depressive symptoms, AOR 2.27; for any mental distress, 2.31). For the hierarchically most severe IPV category of combined physical abuse and severe psychological abuse, there was a substantial increase in the strength of associations for all mental health indices (for depressive symptoms, AOR 4.30; for PTSD symptoms, AOR 3.24; for psychological distress, AOR 5.32; and for any mental distress, AOR 4.51).

Because of cell size restrictions, the second multiple logistic regression analysis examining the composite index of TEs of conflict and IPV focuses on depressive symptoms and any mental distress. Table 3 shows that within the composite no abuse/low regard/respect grouping, there was a regular increase in prevalence of depressive symptoms and any mental distress with increasing exposure to TEs of conflict. Where women were exposed to two to three TEs, the AOR for depressive symptoms was 1.99; for any mental distress AOR 1.56. Where there was exposure to four or more TEs, the associations were even stronger (for depressive symptoms, AOR 2.53; for any mental distress, AOR 2.15).

The reference category for all further comparisons comprised women experiencing 0–1 conflict-related TEs and the no abuse or low respect/regard category of IPV. The strength of association with depressive symptoms increased with greater severity of the combined index of TE and IPV exposure. For women experiencing severe psychological abuse and two to three TEs, AOR 2.65; for women with four or more TEs, AOR 3.95; for physical abuse alone and two to three TEs, AOR 2.66; for physical abuse and four or more TEs, AOR 8.16; for combined physical and severe psychological abuse and two to three TEs, AOR 8.59; and for four or more TEs, AOR 9.78.

A similar pattern was found for any mental distress: for severe psychological abuse, for women with two to three TEs, AOR 2.33; for four or more TEs 3.60; for physical abuse alone and two to three TEs, AOR 2.55; for four or more TEs, AOR 7.03; and for combined physical and severe psychological abuse and two to three TEs, AOR 6.64; and for four or more 4 TEs, AOR 10.45.

Combining those with four or more TEs and either physical abuse alone or severe psychological and physical IPV, yielded 184 women (11% of sample). Among these women, 78 (42%) reached threshold for depressive symptoms, and 93 (51%) for any mental distress, representing an ultra-high-risk group.

## Discussion

To the best of our knowledge, our study is the first conducted in a LI-PC country to identify an ultra-high group of pregnant women, defined by exposure to past traumas of conflict and IPV, who manifested a high level of depressive and other stress-related symptoms. Specifically, one in 10 women (11%) were exposed to *⩾*4 TEs and physical IPV (alone or with severe psychological abuse), and of these, two-fifths met threshold for depressive symptoms and one in two for any mental distress.

Prior to discussing the translational implications of our findings, we consider the strengths and limitations of the study. The sample of pregnant women is by far the largest recruited to a mental health study in the LI-PC field to date, and the response rate was high. Recruitment covered all major antenatal clinics in a major administrative district of the country including urban areas in the capital city as well as surrounding rural populations. The cross-sectional nature of our study cautions, however, against drawing definitive causal inferences, for example, between reports of past TEs and current mental distress. Nevertheless, the profile of conflict-related TEs recorded is consistent with the known history of Timor-Leste and with data gathered in our past studies.^31, 32^ Further studies will be needed to test whether our findings can be generalised to other parts of Timor-Leste and other LI-PC countries.

Transcultural error in adapting and applying measures is a perennial risk in the field. We attempted to reduce error by undertaking a rigorous process of cultural consultation, translation and back-translation of measures. Based on advice by cultural consultants, we did not attempt to record sexual abuse in our assessment of IPV. We note, however, that any bias introduced by this omission would have attenuated rather than strengthened the associations we found between IPV and mental distress. We did not assess the possible impact of IPV on women's physical health or on the unborn child, areas of inquiry that warrant future study.

As in previous studies, we found substantial overlap in the types of IPV, underscoring the need to devise a rational hierarchically ordered typology of abuse that includes both psychological and physical forms. The regular dose–effect pattern we found between our IPV categories and mental distress provides substantial support for the utility of our hierarchical approach to assessment. The hierarchy is not intended to imply, however, that the lowest IPV category (low respect/regard) is of little or no significance. It is possible that this pervasive form of abuse is associated with non-clinical levels of distress and, in addition, represents the harbinger of more severe types of IPV in the future life of the woman.^33^ We note further that, based on human rights, gender equity and primary prevention considerations, all forms of IPV warrant social and policy attention.^34^

It is notable that the TEs of conflict occurred either during the Indonesian occupation which ended 14 years prior to our study or in the period of internal conflict that occurred 6 years earlier. Our findings therefore attest to the enduring psychological effects of extensive exposure to conflict-related TEs on women when they enter the child-bearing period in later life, especially if they also have experienced IPV. In relation to IPV, the category of physical violence and severe psychological abuse (threatening, intimidating and controlling spousal behaviours), a pattern affecting one in five women, was associated with a remarkably high prevalence of symptoms of depression and other indices of mental distress (all AORs>3). Our key finding was the regular, dose–effect relationship that emerged between our stratified typology of combined trauma (conflict-related TEs and IPV) and the mental health indices measured. The analysis identified an ultra-high-risk group of women, representing 11% of the sample, who had experienced extreme levels of conflict-related trauma and physical IPV (many also being exposed to severe psychological IPV). Among that group, more than half met criteria for mental distress, a 10-fold increase compared with the reference category.

It is well-recognised that mass conflict constituted one of the major impediments to women in LMICs realizing the Millennium Development Goals.^35^ The specific triad of jeopardy (high exposure to the traumas of mass conflict, IPV, co-occurrence of depression and other stress-related mental health symptoms) identified herein is likely to have played a major role in reducing the capacity of women in the antenatal period from participating fully in programmes of social, economic and political recovery in fragile and resource-deficient LI-PC countries. These observations are particularly relevant to high-fertility LI-PC societies such as Timor-Leste where women in their early and middle adult years spend a considerable period of their time in the perinatal phase. Our findings, therefore, should alert policy-makers to the imperative to promote programmes aimed at identifying and addressing this major public health problem. The first step is to ensure a comprehensive and effective programme of detection of high-risk women in antenatal clinics. The indices we have used (TEs, IPV and mental health) can be abbreviated to facilitate routine screening of women in the clinic setting. Implementing a systematic procedure for collecting and reviewing data will provide the foundation for applying a tiered set of interventions that accurately target the needs of pregnant women.

Given the scarce public health and mental health resources available,^36, 37^ a stratified, stepped care model for intervention would seem most appropriate. For example, for women in the lower risk categories, a low-cost programme of intervention may be offered by providing group sessions aimed at promoting empowerment, encouraging safety planning, engaging social and family support and, where necessary, seeking legal interventions, followed by monitoring over time to assess whether more intensive interventions are needed should the situation deteriorate, for example, where there is an escalation in IPV and/or a worsening of mental health symptoms.

The ultra-high-risk group of women will require a comprehensive model of intervention that is designed in each setting to be socially and culturally appropriate.^38^ There is a growing consensus that reducing violence in the family can only be achieved if men are directly involved in the process.^34, 39^ A recent study conducted in a LI-PC country, Côte d'Ivoire, offers preliminary evidence that a well-designed programme can be effective in challenging men's inequitable gender values and behaviours that justify and condone violence against women.^40^ Engaging men remains a challenge, however, particularly in societies where patriarchal attitudes and the psychological effects of war-related trauma combine to increase risk of male-perpetrated violence in the home.^25, 41^ A graduated approach based on a family intervention model would seem most appropriate for the ultra-high-risk group of women we have identified, particularly given the multifaceted nature of the problems they confront. At the outset, engagement and rapport-building with both members of the couple are of paramount importance, commencing initially with a commitment to non-violence during the process. A psycho-educational intervention will assist in setting the stage by detailing the effects of the stresses of mass conflict and subsequent conditions of adversity on the family, topics that are of mutual interest to men and women. Once rapport is established, the focus can turn to the woman's symptoms of depression and traumatic stress, a key aim being to sensitise the male partner to the impact of environmental stressors, both external and internal to the family, on the woman's emotional well-being.^42, 43^ Counselling can then progress to exploring the sensitive issue of family conflict, and ultimately the deleterious impact of IPV on the woman's health, mental well-being and on the future health of the infant. In the course of the discussion, it is vital to confront issues of male power, tracing its roots to traditional, patriarchal norms, but emphasising the role of personal responsibility in curtailing the behaviour, in keeping with changing values and mores in the society that mandate gender equality and the rights of women, consistent with emerging national policy and international standards and conventions. In the process, the counsellor will also provide practical skills to improve interpersonal communication and the capacity to resolve challenges within the family, without resorting to confrontation or aggression. Achieving a foundation of mutual understanding of the pathways that have led to male-perpetrated violence in the family may act as a motivator for change, particularly when the male recognises the benefits of establishing a culture of respect, harmony and a sense of safety in the home for all the family.^42^ The comprehensive model also recognises that, in a minority of ultra-high-risk women, symptoms of depression or other stress-related disorders may be so severe or disabling that referral to mental health services will be needed for specialised psychotherapeutic interventions, and in some instance, the judicious use of antidepressant medications.

When initiating any intervention, it is vital for the counsellor to remain vigilant to the risk of recurrent or escalating IPV, particularly retaliatory violence enacted by the male in response to his partner's disclosure of IPV to outsiders. Timely sharing of knowledge about domestic violence legislation (which has been implemented recently in Timor-Leste) is important to ensure that the male partner is aware of existing sanctions and mechanisms within the criminal-justice system to deal with perpetrators. In addition, women must be fully informed of the imperative to make contact with emergency domestic violence services and the police if there is threat of further violence. Given the early phase of research focusing on interventions in this field, future studies will need to evaluate the feasibility and effectiveness of different models of family counselling for the constellation of problems we have identified.

In outlining this optimal care model for LI-PC countries, we recognise that any intervention must be culturally sensitive, contextually feasible and sustainable. Lack of resources, a limited skills base and access to supervision present major challenges to developing a programme of intervention such as the one we have outlined. Persisting patriarchal values may also create resistance within the community to gender equity-based programmes of this type. Other forms of resistance may occur at multiple levels within the system; in services, because providers do not wish to alienate men or provoke a situation where males prevent their female partners from receiving antenatal care; and among policy-makers because of the persistence of unarticulated but deep-seated gender biases in some officials. There is evidence of progress, however, in achieving momentum in developing gender equity-based programmes in LI-PC countries. Timor-Leste has formulated and adopted gender equity policy frameworks, legislation has been introduced to prevent IPV, and services have been established to protect and assist women affected by this pervasive form of abuse.^44^

## Figures and Tables

**Table 1:** Descriptive statistics for socio-demographic characteristics and mental health indices

*Characteristics*	*Total number of women (*n=*1672)*	*Col %*
*Age groups <20 years*	141	8.4
20-24	568	34.0
25-29	576	34.4
30-34	273	16.3
⩾35	114	6.8

*Marital status*		
Married	419	25.1
Engaged/living together	1240	74.2
Separated/divorced	9	0.7

*Educational status*		
None or Primary school	271	16.2
Junior/Senior High School	961	57.5
Technical College/Diploma	104	6.4
University	336	20.1

*Occupation*		
Unemployed	949	56.8
Student	180	10.8
Farming/small trade	200	12.0
Government/non-government organisation	190	11.4
Other	153	9.2

*Exposure to categories of war-related traumatic events*^b^		
0	183	11.0
1	273	16.3
2 or 3	602	36.0
⩾4	613	36.7

*Intimate partner violence (IPV) derived groups^a^*		
No abuse at all	180	10.8
Low respect/regard and no abuse	550	32.9
Severe psychological abuse (threatening, intimidation and controlling)	511	30.6
Physical abuse only	103	6.2
Physical abuse+severe psychological abuse	327	19.5

*Mental health indices*		
Edinburgh Depression Scale (EPDS): threshold ⩾13.0	329	19.7
Post-traumatic Stress Disorder (PTSD): threshold ⩾2.0	96	5.7
Kessler-10 (K10) psychological distress: threshold ⩾30.0	105	6.3
Any mental distress (threshold for at least one of EDS, PTSD or K10)	403	24.1

Abbreviation: TE, traumatic event.

a There was one missing participant for TEs; and one missing for IPV and these two have been excluded from further analyses leaving an analytic sample of *n*=1671.

b IPV items are grouped as follows.

Low respect/regard only: participants included if endorsed one or more items from this group but not from forms of IPV categorised higher in the hierarchy. Example of items include spends his free time with you; consults on different household matters with you; respects you and your wishes; does not trust you with any money.

Severe psychological (threatening, intimidating and controlling): participants included if endorsed one or more items from this group. Participants may have endorsed items from low respect/regard but were not included if endorsed any physical abuse items. Items included in this group are: he is affectionate with you; jealous or angry if you talk to other men; frequently accuses you of being unfaithful; does not permit you to meet your girl friends; tries to limit your contact with your family; insists on knowing where you are at all time; humiliates you in front of others; threatens you/someone close to you with harm.

Physical violence: participants were included if they endorsed one or more of: pushes you, shakes you or throws something at you; slaps you or twists your arm; punches you with something that could hurt you; kicks/drags you; tries to strangle/burn you; threatens you with a knife, gun or other type of weapon; attacks you with a knife, gun or other type of weapon; other ways your husband hurts you. Participants were included in the physical violence only group if they did not endorse any items from the threatening/jealous/controlling group. They may have endorsed items from the low respect/ regard category.

Physical violence plus severe psychological (threatening, intimidating and controlling): participants were included in this group if they endorsed a minimum of one item from physical violence and one from threatening/jealous/controlling. In addition, they may have endorsed items from lack of sharing/respect.

**Table 2:** Multivariate associations between categories of IPV and mental health indices

*Intimate partner violence (IPV) categories*		*Edinburgh depression scale (EDS)*		*Post-traumatic stress disorder (PTSD)*		*Kessler-10 (K10) psychological distress*		*Any mental distress (threshold for at least one of EDS, PTSD or K10)*	
		*EPDS⩾3.0*		*PTSD⩾2.0*		*K10⩾30.0*			
	*Total*	*Row % (*n*)*	*Adjusted odds ratio (95% CI)*	*Row % (*n*)*	*Adjusted odds ratio (95% CI)*	*Row % (*n*)*	*Adjusted Odds ratio (95% CI)*	*Row % (*n*)*	*Adjusted odds ratio (95% CI)*
Combined no IPV and low respect/regard alone (ref. category)	730	11.8 (86)	1.00	3.6 (26)	1.00	3.4 (25)	1.00	14.9 (109)	1.00
Severe psychological abuse alone^a^	511	18.0 (92)	1.61 (1.17–2.23)^b^	4.7 (24)	1.26 (0.71–2.24)	3.7 (19)	1.09 (0.59–2.03)	22.9 (117)	1.67 (1.24–2.24)^c^
Physical abuse alone^a^	103	25.2 (26)	2.27 (1.37–3.77)^c^	7.8 (8)	2.20 (0.96–5.04)	6.8 (7)	2.08 (0.87–5.01)	30.1 (31)	2.31 (1.45–3.71)^c^
Severe psychological and physical abuse^a^	327	38.2 (125)	4.30 (3.12–5.96)^c^	11.6 (38)	3.24 (1.90–5.50)^c^	16.5 (54)	5.32 (3.20–8.87)^c^	44.6 (146)	4.51 (3.34–6.09)^c^
Column total	1671	19.7 (329)		5.7 (96)		6.3 (105)		24.1 (403)	
*χ2*-test		*P*<0.001		*P*<0.001		*P*<0.001		*P*<0.001	

Abbreviation: CI, confidence interval; IPV, intimate partner violence.

There was only one missing case across all 20 IPV items and that case has been excluded from the analyses (*n*=1671).

a Note: 401 persons in the severe psychological abuse alone category, 81 in the physical abuse alone category and 271 in the combined severe psychological and physical abuse category endorsed at least one item in the low respect/regard group.

b Adjusted odds ratios (AORs) are significant at *P*<0.05 as compared with reference category.

c AORs are significant at *P*<0.01 as compared with reference category.

**Table 3:** Associations of combined categories including IPV and conflict-related trauma exposure with mental health indices

*Combined IPV and conflict-related traumatic event categories*		*Total*	*Edinburgh Depression Scale (EDS): EDS⩾13.0*			*Any mental distress (threshold for at least one of EDS, PTSD or K10)*		
			n	*%*	*AOR (95%CI)*	n	*%*	*AOR (95%CI)*
Combined no IPV and lack of sharing/respect (reference category for all further comparisons)	0 to 1 trauma categories	217	17	7.8	1.00	24	11.1	1.00
	2–3	256	32	12.5	1.99 (1.06–3.73)^a^	38	14.8	1.56 (0.90–2.71)
	*⩾*4	257	37	14.4	2.53 (1.36–4.70)^b^	47	18.3	2.15 (1.25–3.68)^a^
*χ2*-test			*P*=0.080			*P*=0.088		
Severe psychological abuse alone	0 to 1	137	22	16.1	2.28 (1.15–4.51)^a^	27	19.7	1.99 (1.09–3.64)^a^
	2–3	202	33	16.3	2.65 (1.41–4.98)^b^	42	20.8	2.33 (1.34–4.04)^b^
	*⩾*4	172	37	21.5	3.95 (2.10–7.40)^b^	48	27.9	3.60 (2.08–6.23)^b^
*χ2*-test			*P*=0.339			*P*=0.154		
Physical abuse alone	0 to 1	28	4	14.3	1.90 (0.58–6.18)	5	17.9	1.71 (0.59–4.93)
	2–3	38	7	18.4	2.66 (1.01–7.00)^a^	9	23.7	2.55 (1.08–6.05)^a^
	*⩾*4	37	15	40.5	8.16 (3.53–18.85)^b^	17	45.9	7.03 (3.23–15.29)^b^
*χ2*-test			*P*=0.026			*P*=0.028		
Severe psychological and physical abuse	0 to 1	75	19	25.3	3.86 (1.86–7.99)^b^	23	29.3	3.34 (1.73–6.41)^b^
	2–3	105	43	41.0	8.59 (4.52–16.32)^b^	46	43.6	6.64 (3.72–11.83)^b^
	*⩾*4	147	63	42.9	9.78 (5.31–18.02)^b^	78	53.1	10.45 (6.06–18.01)^b^
*χ2*-test			*P*=0.031			*P*=0.003		

Abbreviations: AOR, adjusted odds ratio; CI, confidence interval; IPV, intimate partner violence; K10, Kessler-10; PTSD, post-traumatic stress disorder.

The reference category for all analyses in table is persons in the combined group of no IPV or low sharing/respect only who have experienced 0–1 traumatic events (*n*=217). Note that women assigned to severe psychological abuse and physical abuse categories (and combination) may have endorsed items of the low sharing/respect form of psychological abuse. *P-*values from *χ2*-test indicate significance in the dose–effect trend for increasing exposure categories of TEs and higher prevalence in relevant mental health indices.

a Adjusted Odds ratios (AORs) are significant at *P*<0.05 as compared with reference category.

b AORs are significant at *P*<0.01 as compared with reference category.
